# Calcium and Microhardness Quantification in Healthy and Fluorotic Dentin Conditioned with a Self-Etching System: An In Vitro Study

**DOI:** 10.3390/dj13040168

**Published:** 2025-04-17

**Authors:** José Alejandro Rivera Gonzaga, Ana Josefina Monjarás Ávila, Louis Hardan, Norma Verónica Zavala Alonso, Carlos Enrique Cuevas Suárez, Nicolas Nassar, Ahmed A. Holiel, Naji Kharouf, Youssef Haikel, Rim Bourgi

**Affiliations:** 1 Dental Materials Laboratory, Academic Area of Dentistry, Autonomous University of Hidalgo State, San Agustín Tlaxiaca 42160, Mexico; jose_rivera10098@uaeh.edu.mx (J.A.R.G.); ana_monjaras@uaeh.edu.mx (A.J.M.Á.); cecuevas@uaeh.edu.mx (C.E.C.S.); 2 Department of Restorative Dentistry, School of Dentistry, Saint-Joseph University, Beirut 1107 2180, Lebanon; louis.hardan@usj.edu.lb; 3 Department of Digital Dentistry, AI, and Evolving Technologies, Faculty of Dental Medicine, Saint Joseph University, Beirut 1107 2180, Lebanon; nicolas.nassar.dds@hotmail.com; 4 Faculty of Dentistry, Autonomous University of San Luis Potosí, San Luis Potosí 78000, Mexico; nveroza@fest.uaslp.mx; 5 Department of Orthodontics, Faculty of Dental Medicine, Saint Joseph University, Beirut 1107 2180, Lebanon; 6 Craniofacial Research Laboratory, Faculty of Dental Medicine, Saint Joseph University of Beirut, Beirut 1107 2180, Lebanon; 7 Conservative Dentistry Department, Faculty of Dentistry, Alexandria University, Alexandria 21532, Egypt; ahmed.holiel@alexu.edu.eg; 8 Department of Restorative Sciences, Faculty of Dentistry, Beirut Arab University, Beirut 115020, Lebanon; 9 Department of Biomaterials and Bioengineering, INSERM UMR_S 1121, University of Strasbourg, 67000 Strasbourg, France; dentistenajikharouf@gmail.com (N.K.); youssef.haikel@unistra.fr (Y.H.); 10 Department of Endodontics and Conservative Dentistry, Faculty of Dental Medicine, University of Strasbourg, 67000 Strasbourg, France; 11 Pôle de Médecine et Chirurgie Bucco-Dentaire, Hôpital Civil, Hôpitaux Universitaire de Strasbourg, 67000 Strasbourg, France

**Keywords:** calcium, dentin, fluorosis, microhardness, self-etch system

## Abstract

**Background:** Dental fluorosis can affect the micromorphology of dentin, a fact that could present constraints relating to the structural, mechanical, and chemical stability of dentin when it is demineralized in operative maneuvers. **Introduction:** The aim of this article is to quantify the amount of calcium and the microhardness of both a healthy and a fluorotic dentin through conditioning with a two-step self-etching system (Optibond Versa, Kerr, CA, USA). **Methods:** Dentin samples were obtained from healthy molars diagnosed with mild, moderate, and severe fluorosis. The amount of calcium was quantified utilizing an atomic absorption spectrophotometer. The hardness was evaluated using a Vickers durometer. Two dentin samples from each study group were examined using scanning electron microscopy (SEM). **Results:** A one-way analysis of variance (ANOVA) and Tukey–Kramer test were applied as post hoc tests for determining the differences in calcium values between the study groups and to show the difference in the hardnesses evaluated. The Student’s t-test was applied to related samples. The level of significance was set at *p* < 0.05. Statistically significant results were obtained for the amount of calcium and microhardness of the healthy dentin group. The SEM images demonstrated irregular etching patterns in the fluorotic dentin, indicating potential bonding challenges. **Conclusion:** In conclusion, there is a lower amount of calcium and a significant reduction in microhardness in healthy dentin when applying the self-etching system compared to fluorotic dentin. Fluorotic dentin is more resistant to demineralization, which may influence adhesive bonding strategies. Clinicians should consider adjusting conditioning protocols for optimal adhesion in fluorotic teeth.

## 1. Introduction

Numerous investigations have been carried out that attempt to explain the microstructural changes in dental tissue when exposed to different adhesives [1,2,3,4]. Due to the action of acids that promote dental conditioning prior to adhesion, dentin undergoes alterations in its inorganic components [5]. These acids cause the demineralization of the tissue and the consequent loss of mineral crystals, which lead to an alteration in the network of collagen fibers, a vital process in the formation of a stable hybrid layer in the long-term. Because of these effects, a decrease in sensitivity and marginal microfiltration is expected, which translates into a lower recurrence of decay and failures in cosmetic restorations [6].

It is essential to bear in mind that conditioned dentin displays unique histological characteristics, as it experiences denaturation due to demineralization and the eventual breakdown of the collagen polymeric network when exposed to acidic environments [7]. Some studies show that the deepest areas of the dentin altered by an acid application must contain residual minerals, particularly intrafibrillar minerals, to allow nucleation and growth during physiological remineralization [6,8]. If the remaining crystallites are not sufficient, remineralization may not take place since the mineral content influences the characteristics of the subsequent remineralization. A remineralized tissue which has had its mechanical properties restored under physiological conditions is an indication that mineral crystallites are closely associated or even chemically linked to the collagen matrix [9]. The quality of dentin, defined by its microstructure, mineral density, and the spatial distribution of minerals within the organic matrix, plays a key role in the success of adhesive restorations [7].

The analysis of microhardness is an important aspect of assessing the mechanical stability of the dentin after the application of an adhesive, as it reveals the degree of demineralization, its effectiveness, and the durability of the bonded interfaces. Since self-etch systems result in varying degrees of demineralization, evaluating the microhardness aids in forming accurate perceptions regarding the adhesive–dentin interaction and predicting the long-term performance of the restorations [6,8,10]. Knowing that fluorotic dentin already displays compositional and structural changes, excessive dentin demineralization may compromise the stability of the bond, proving that microhardness assessment is a valuable process in assessing the effectiveness of an adhesive [11,12].

It has been reported that dental fluorosis affects the micromorphology of dentin [11], a fact that could pose limitations in terms of structural, mechanical, and chemical stability when demineralized in operative maneuvers [12]. When applying self-etch systems to dentin, which is less aggressive for the pulp–dentin complex [5,13], it is expected that the smear layer and detached minerals are reincorporated into the hybrid layer [14] and thus the mechanical integrity of the dentin seems to be restored, although this is only partial because the mineral is reincorporated into the structure, binding to the matrix differently from the physiological one [7].

The bonding performance of self-etch adhesives is linked to the presence of functional monomers, which interact with hydroxyapatite (HAp) and calcium ions at the adhesive interface [1]. The Adhesion–Decalcification (A–D) concept states that, initially, acidic functional monomers bind ionically to calcium in HAp. The stability of this bond influences the extent of demineralization: if stable, it contributes to a durable adhesion; if unstable, further calcium dissolution occurs, which weakens the hybrid layer. A key functional monomer, 10-methacryloxydecyl dihydrogen phosphate (10-MDP), forms stable calcium–phosphate complexes, enabling chemical bonding to dentin and improving long-term adhesion durability [2,8].

Due to the necessary demineralization processes to which dentin is subjected during the adhesion process [15], it is crucial to determine the degree of calcium loss associated with self-etching systems. If this is linked to the loss of microstructural microhardness in healthy and fluorosis-affected dentin, the results contribute to a better understanding of the interaction between self-etching systems and the dentinal substrate. This knowledge helps recompose the complex structure of dentin and restore its mechanical behavior against masticatory forces, providing valuable insights for the field of restorative dentistry. Accordingly, the null hypothesis tested that the self-etching adhesive system does not cause a significant difference in calcium loss or microhardness reduction between healthy and fluorosis-affected dentin.

## 2. Materials and Methods

### 2.1. Collection and Preparation of Samples

Eighty human molars, extracted within a month prior to this study, were collected from clinics in three areas of San Luis Potosí, Mexico: (1) Ciudad Valles (0.1 and 0.6 ppm F), (2) San Luis Potosí City (0.7 and 2 ppm F), and (3) Salitral de Carrera (2 and 5 ppm). The teeth were stored in a 0.2% thymol solution and subsequently cleaned using ultrasound (Cavitrón, Varios 350 NSK, IL, Cuyahoga, Falls, OH, USA). After cleaning, they were placed in an ultrasonic bath (Biosonic UC300-115B, Coltene/Whaledent, Cuyahoga, Falls, OH, USA) with glutaraldehyde for 15 min to remove debris and impurities. Finally, they were dried and analyzed by visual observation to determine the severity of fluorosis according to the Fejeskov index, and they were classified as healthy (H) or having mild (L), moderate (M), or severe (S) fluorosis.

**Figure 1:**
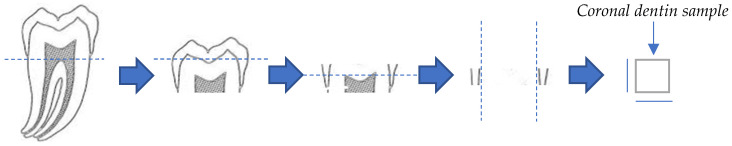
Diagram showing the cuts made to obtain coronal dentin samples.

All molars were stored in distilled water (Milli-Q, Millipore Co., Billerica, MA, USA) at 4 ° C until the experimental procedures were performed. A cross-section was made at the level of the cement–enamel junction using a low-speed diamond disk with constant irrigation (# 7910, medium grain; Brasseler, Savannah, GA, USA) to obtain the dentin samples. Then, the occlusal and proximal enamel were removed from the samples to obtain 3 mm dentin blocks, as shown in Figure 1.

The dentinal surface of the blocks was flattened with 600 silicon carbide (SiC) paper and cooled with water to produce a uniform smear layer. Each block was weighed on a precision scale (BL6OS, Sartorius, Bradford, MA, USA) to standardize the weight of each sample (*n* = 12 per subgroup). Then, a self-etching protocol was subsequently applied (Table 1).

### 2.2. Atomic Absorption Spectrometry Test

Initially, five standard samples were prepared for the calcium calibration curve. Lanthanum nitrate (0.5%, lanthanum nitrate hexahydrate: (NO_3_)_3_·6H_2_O) was added to eliminate interference from other ions. Afterwards, the sample was placed in a plastic container, and the self-etching technique was applied, as shown in Table 1. Following this, the container was filled with 5 mL of deionized water. These samples were kept under constant agitation before the test was carried out to homogenize the calcium extracted in the solution. A blank was made with deionized water to determine the calcium levels in the absence of the treated sample. Finally, the solutions for each study group were applied to the Atomic Absorption Spectrophotometer (AAS; AAnalyst 400, Perkin Elmer Analytical Instruments, mod. 5100ZL Waltham, MA, USA) to analyze the release of calcium from each tooth, using the flame technique with a high purity air–acetylene flame (6 mA, 422.7 nm, 0.5 mm; Shimadzu Corporation, AA-680, Tokyo, Japan). Considering that spectrometry is a conventional method, the use of three repetitions was decided. Readings were expressed in parts per million (ppm).

### 2.3. Vickers Microhardness Test

Twenty dentin samples for each study group were mounted on acrylic blocks to support testing under the indenter. The dentin surface was flattened using 600-grit SiC paper while being cooled with water to create a uniform smear layer. All samples underwent three hardness indentations before and after applying each experimental technique. The indentations were performed using a Vickers HV-1000 hardness tester (DongGuan Sinowon Precision Instrument Co., Ltd., South District, Dongguan, China) with a 50 gf load and a residence time of 30 s, maintaining a 0.5 mm distance between each indentation. The average value was recorded as the surface microhardness for each study group.

### 2.4. Scanning Electron Microscopy Evaluation

Two dentin samples from each study group were selected for observation. The self-etch primer was applied as specified in Table 1. The samples were then sequentially immersed in increasing concentrations of ethanol (30%, 40%, 50%, 60%, 70%, 80%, and 90%) for 30 min each, followed by 100% ethanol for 24 h. Next, they were fixed onto the specimen holder using double-faced stickers and coated with a thin layer of gold using a sputter coater (S150A, Edwards, London, UK) to enhance electrical conductivity. The samples were examined using a scanning electron microscope (SEM) (JEOL JSM-6510, Tokyo, Japan), operated at 10 kV and ×300 magnification.

### 2.5. Statistical Analysis

Statistical analysis was performed using the Statistical Package for Social Sciences (SPSS) version 20.0 (Chicago, IL, USA). Data were presented as the average ± standard deviation. The groups were compared using a one-way analysis of variance (ANOVA); the Tukey–Kramer post hoc test assessed the significance of the difference in means within and between the groups after determining normality through the Shapiro–Wilks test or the Brown–Forsythe test and the homogeneity of the variations using the Levene test. The Student’s t-test was applied to related samples to determine the difference in the evaluated hardness. A *p* < 0.05 was considered statistically significant.

### 2.6. Ethical Considerations

This article does not contain any studies involving human or animal participants conducted by any of the authors. The patient’s consent to donate each tooth for the study was considered. All the experiments performed were approved by the guidelines of the Ethics Committee of the Institute of Health Sciences, the Autonomous University of the State of Hidalgo, registration number CEEI-032-2019, and adhered to the principles of the Declaration of Helsinki.

## 3. Results

Table 2 reveals the averages and standard deviation in the calcium ion quantification and microhardness of healthy dentin with different degrees of fluorosis.

**Figure 2:**
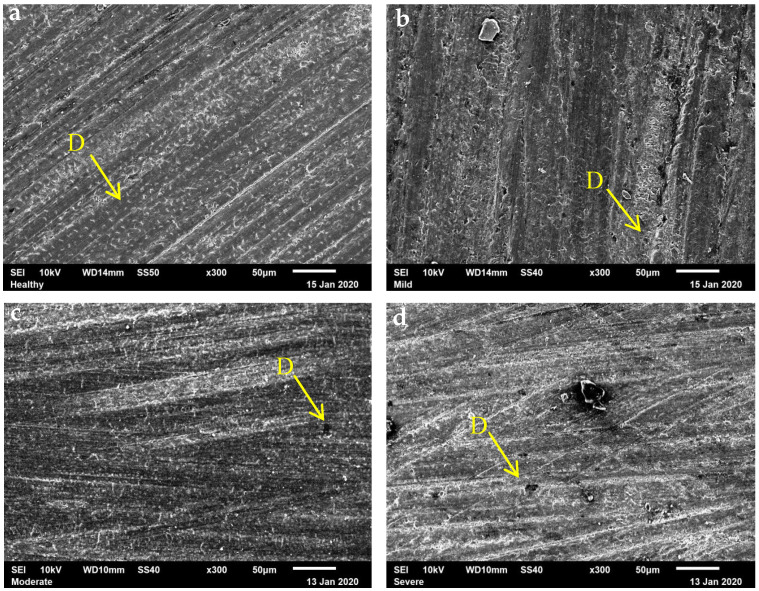
Scanning electron microscopy (SEM) ×300 images of the micromorphology of healthy dentin and dentin with mild, moderate, and severe fluorosis conditioned using the OptiBond Versa self-etch primer showing the surface micromorphology of healthy dentin (**a**), dentin with mild (**b**), moderate (**c**), and severe (**d**) fluorosis conditioned with the self-etching primer. (**a**): Small dentinal tubules covered by smear layer can be seen on a homogeneous surface, (**b**): Some exposed dentinal tubules can be seen on the tooth surface that are larger than those in healthy dentin, with a disorganized microstructure, (**c**): Thick smear layer completely covers the dentin surface, obscuring the dentinal tubules, (**d**): Large dentinal tubules and a surface with craters can be seen on a disorganized substrate. D: Dentinal tubules.

Microphotographs of healthy dentin and fluorosis-affected dentin treated with the self-etching adhesive OptiBond Versa are presented in Figure 2. Healthy dentin exhibited a homogeneous surface with small dentinal tubules covered by a smear layer. Mild-fluorosis dentin displayed larger dentinal tubules and a more disorganized microstructure. In moderate-fluorosis dentin, the presence of a thick smear layer obscured the dentinal tubules. Severe fluorosis dentin showed a highly disorganized substrate with large dentinal tubules and crater-like surface irregularities. These findings indicate that the severity of fluorosis influences the micromorphological response of dentin to self-etching treatment.

## 4. Discussion

With the advent of adhesive dentistry and the application of new and enhanced dentin materials, it is essential that professionals are aware of the importance of good conditioning, as well as the microstructural changes in different substrates exposed to demineralization due to the use of a self-etching primer.

It is well known that dental fluorosis is caused by the high consumption of fluoride at the stage of dental germ formation [16]. The affected enamel presents a superior surface porosity, hypo-mineralization, and a larger amount of proteins retained in the dental enamel during the formation of the dental organ, which cause this type of porosity with this pathology [17]. On the other hand, in dentin, micro-morphological changes occur due to hyper-mineralization, elongated dentinal tubules, and an increased intertubular space. These changes are considered a biomarker of dental fluorosis [18,19].

This study discloses how the amount of calcium is altered when applying a self-etching system to both healthy and fluorotic dentin. The evaluation of calcium ion release in healthy and fluorosis-affected dentin samples was conducted using atomic absorption spectroscopy, a reliable method for quantifying minerals in crystalline structures [20]. Additionally, microhardness measurements were performed before and after the application of the self-etch primer. Given the statistically significant differences observed in both calcium ion and microhardness between healthy and fluorosis-affected dentin following self-etch adhesive application, the null hypothesis was rejected.

Previous studies have reported a high failure rate of resin-based restorations, with recurrent caries being the primary reason for restoration replacement [21,22]. While it is well established that teeth with mild fluorosis are more resistant to decay and teeth with moderate or severe fluorosis are more susceptible to caries due to the damage to the structural integrity of the dental organs [23], fluorosis also negatively impacts adhesive performance, compromising the bonding effectiveness of restorative materials [18].

When healthy dentin is compared with fluorosis-affected dentin, the loss of calcium is significantly lower in the healthy dentin, whereas fluorosis-affected dentin displays nearly three times the amount of calcium loss. Despite this, the microhardness values are inferior in healthy dentin when compared to fluorotic dentin. A significant reduction in microhardness was witnessed in the healthy dentin after the conditioning process, whereas, in the fluorosis-affected dentin, the difference was not statistically significant. This proves that despite the greater calcium loss, the hyper-mineralization characteristics of fluorotic dentin [23] affect its hardness, possibly due to the presence of fluorapatite crystals. Although fluorosis-affected dentin undergoes a significant calcium loss, the presence of fluoride seems to compensate for this issue. In contrast, healthy dentin undergoes a more noticeable structural and mechanical disparity when mineral loss occurs. This disruption in the mineral equilibrium might quicken the failure of the restoration if adequate hybrid layer formation and restoration placement are not guaranteed, particularly due to the vulnerability of the substrate to electrochemical changes brought forth by demineralization [24].

On the other hand, two-step self-etching systems require an initial primer followed by an adhesive, facilitating dentin demineralization and conditioning without the need for rinsing. This allows the dentin to retain the ions released during the demineralization process. These ions are reincorporated into the hybrid layer, contributing to the hybridization process that is essential for enhanced adhesion. It has been suggested that calcium plays a crucial role in improving the interaction between functional monomers and the adhesive system [25], thereby enhancing the material’s performance. This increase in calcium ions strengthens the bonds, providing better chemical adhesion [26], which may be particularly beneficial for dentin affected by fluorosis.

The SEM images reveal a dental substrate with a significant amount of dentin smear on a homogeneous surface in the case of healthy dentin samples, whereas the dentin samples with fluorosis show an irregular surface with craters and larger dentinal tubules that are not completely covered by the smear layer. This observation aligns with a previous study, which disclosed a micromorphology of dentin with larger intertubular spaces compared to normal conditions [27]. This suggests that more time is required for demineralization to achieve reliable results on a non-homogeneous surface with a higher mineral content [18,19]. According to the results of this research, dentin with mild and severe fluorosis exhibits greater calcium loss values than the other groups. This is evident in the images, where a higher number of exposed dentinal tubules were present that could not be fully covered by the smear layer formed during demineralization. This incomplete coverage leads to a lower absorption load during the microhardness test, resulting in higher values. In contrast, healthy dentin, which maintains a more substantial dentin smear layer, distributes the weight more evenly, leading to lower microhardness results [28]. While this may seem less favorable, it actually reflects the normal response to the demineralization process, facilitating better hybridization and providing more calcium ions to interact with the functional monomers of the adhesive, ultimately enhancing bonding performance.

The restoration of the functionality of the decalcified dentin generates mineral ions that are within its composition, such as calcium, phosphate, magnesium, and fluoride [29], that bind and require not only the formation of extra-fibrillary minerals, but also intra-fibrillary ones, within the zones of collagen fibrils [30]. Therefore, intrafibrillar remineralization is crucial for the restoration of the mechanical properties of dentin. Functional monomers in adhesive systems can enhance bonding by improving wetting and demineralization through chemical bonding to calcium [25,28].

Based on the concept of (A–D), the chemical bond potential was evaluated by quantifying calcium dissolution in dentin with varying degrees of fluorosis. The results revealed that a higher calcium dissolution was associated with a larger loss of microhardness in dentin affected by fluorosis, while healthy dentin, with lower calcium dissolution, exhibited a greater loss of microhardness in its structure. This analysis helps to elucidate the underlying mechanism, where carboxylic groups in the adhesive monomers replace the calcium ions in the substrate, forming ionic bonds with the calcium in fluorapatite. This process provides deeper histological insights into the vulnerability of fluorosis-affected dentin. The demineralization process, initiated by an acid that conditions the dentin, plays a critical role in ensuring the stability of restorations over time, enabling them to maintain functionality within the oral environment [29,30,31].

Similar studies have highlighted the impact of fluoride exposure on dentin microhardness, with some studies indicating that higher fluoride concentrations can reduce dentin hardness, while others have demonstrated that remineralization agents can restore hardness in fluorosis-affected enamel and dentin [32,33,34,35].Therefore, understanding the effects of fluoride on dentin, including the chemical bonding and ion exchange processes, is crucial for improving adhesive strategies in restorative dentistry, particularly for fluorosis-affected dentin.

Currently, dentin adhesion continues to be a challenge in restorative dentistry, due to its high inorganic content. When performing demineralization with acidic media, dentin undergoes a denaturation process that impacts all of its microstructural components, restoring itself with the formation of the hybrid layer. This study provides quantifiable data on the extent of mineral loss from samples treated with a self-etch primer, associating it with its microhardness. The value obtained represents the degree of efficiency of this two-step system when performing demineralization on healthy and fluorosis-affected dentin, which permits a stronger knowledge of the micromorphological characteristics of dentin with this pathology and a broader clinical scope for the prognosis of restorations.

## 5. Conclusions

This study highlights the differences in calcium ion release and microhardness alterations between healthy and fluorosis-affected dentin when subjected to self-etching adhesives. Healthy dentin exhibited lower calcium ion release but experienced a greater reduction in microhardness after conditioning. In contrast, fluorosis-affected dentin showed higher calcium ion release but demonstrated greater resistance to microhardness loss.

These findings suggest that fluorotic dentin is less affected by demineralization but may present challenges in adhesive bonding due to its altered mineral composition and structural characteristics. The differences observed in bonding performance emphasize the need for tailored adhesive protocols when working with fluorotic dentin to optimize adhesion and long-term restoration success. Further studies should explore alternative etching strategies and their impact on adhesion effectiveness in fluorotic substrates.

## Figures and Tables

**Table 1:** Self-etching technique and application indications.

Technique	System	Content	Indications
Self-etch primer application	OptiBond Versa (Kerr, CA, USA)	Primer Monomers: glycerol phosphate dimethacrylate (GPDM). Hydrophilic comonomers including mono and difunctional methacrylate monomers. Solvents: water, acetone, and ethyl alcohol. Photoinitiator: camphorquinone (CQ) based. Adhesive Monomers: hydrophobic, structural, and cross-linking monomers. Solvents: ethyl alcohol. Photoinitiator: CQ based. Fillers: 0.4-micron barium glass nano-silica. Fluoride: sodium hexafluorosilicate.	Apply with a micro brush for 20 s and then dry with oil-free air for 5 s.

**Table 2:** Averages and standard deviation in calcium ion quantification and microhardness of healthy dentin with different degrees of fluorosis.

Fluorosis Degree	Amount Ion Calcium Mean ± SD	Sig.		Hardness Before Mean ± SD	Hardness After Mean ± SD	Sig.
Healthy	0.343 ± 0.361	Mild	0.000	53.95 ± 2.37	49.34 ± 5.57	0.030
		Moderate	0.009			
		Severe	0.001			
Mild	1.045 ± 0.215	Healthy	0.000	56.54 ± 5.20	51.67 ± 7.92	0.107
		Moderate	0.524			
		Severe	0.961			
Moderate	0.840 ± 0.552	Healthy	0.009	58.76 ± 3.68	53.70 ± 4.39	0.263
		Mild	0.524			
		Severe	0.814			
Severe	0.972 ± 0.237	Healthy	0.001	60.17 ± 4.97	60.07 ± 1.93	0.979
		Mild	0.961			
		Moderate	0.814			

Fluoride quantity data in ppm. Microhardness data in Vickers. Sample per group *n* = 12. Statistical significance *p* > 0.05. SD: standard deviation.

## Data Availability

Dataset available on request from the authors.
